# Biofilm dynamics under salt exposure: insights from irrigation piping systems

**DOI:** 10.1093/ismeco/ycag001

**Published:** 2026-01-08

**Authors:** Yan Wang, Pengfei Hu, Han Yu, Alex Furman, Olivier Habimana

**Affiliations:** Faculty of Civil & Environmental Engineering, Technion-Israel Institute of Technology, Haifa 3200003, Israel; Environmental Science and Engineering Research Group, Guangdong Technion-Israel Institute of Technology, Shantou 515063, China; Institute of One Health Science, School of Civil & Environmental Engineering and Geography Science, State Key Laboratory for Quality and Safety of Agro-products, Ningbo University, Ningbo 315211, China; Biotechnology and Food Engineering Program, Guangdong Technion-Israel Institute of Technology, Shantou 515063, China; Faculty of Civil & Environmental Engineering, Technion-Israel Institute of Technology, Haifa 3200003, Israel; Environmental Science and Engineering Research Group, Guangdong Technion-Israel Institute of Technology, Shantou 515063, China; Biotechnology and Food Engineering Program, Guangdong Technion-Israel Institute of Technology, Shantou 515063, China; Faculty of Biotechnology and Food Engineering, Technion-Israel Institute of Technology, Haifa 3200003, Israel

**Keywords:** biofilm, salinity, irrigation water, extracellular polymeric substances (EPS), microbial community, meta-transcriptomics

## Abstract

Global agricultural dependence on blended saline and freshwater irrigation mandates a mechanistic understanding of how salinity influences microbial biofilms within distribution networks, which are pivotal mediators of water quality and pathogen viability. Here, we examine the architectural, mechanical, and operational reactions of multi-species biofilms to saline exposure (0.6% NaCl) utilizing a regulated laboratory-scale irrigation model. Through a cohesive methodology combining confocal microscopy, atomic force microscopy, 16S rRNA sequencing, and meta-transcriptomics, we elucidate that salinity instigates a pivotal trade-off in biofilm maturation. While salt stress consistently suppressed live and dead cell biovolumes, it induced a significant enhancement of extracellular polymeric substances (EPS), leading to a thicker, EPS-rich biofilm architecture. These saline biofilms exhibited a lower adhesive force and Young's modulus, indicating a softer and less sticky surface. A community analysis revealed a reduction in taxonomic heterogeneity, along with an increase in specialized taxa associated with hydrocarbon decomposition functionalities, such as *Hydrogenophaga* and *Nakamurella*. Consequently, transcriptomic characterization revealed substantial upregulation of genes implicated in lipid distribution, ionic equilibrium, and oxidative stress mitigation, in conjunction with a downregulation of intercellular adhesion pathways. Our findings reveal that salinity drives biofilm adaptation towards a protected, EPS-dominated state with a functionally specialized community, suggesting a potential increase in the resilience of biofilms and risk of pathogen shielding in saline irrigation systems.

## Introduction

The worldwide deficiency of water presents a substantial obstacle for agricultural practices, and it is forecasted that between 2019 and 2050, the volume of food consumption is expected to increase by 60% [1, 2]. This growing pressure on already scarce and available freshwater resources is further exacerbated by the concomitant problem of soil salinization that affects about 25% of global irrigated land, thus reducing crop productivity and compromising long-term ecosystem stability [3]. As such, the use of saline and freshwater mixtures has become a promising technique for achieving sustainable water resources utilization under high-yield cropping conditions [4–6]. However, poor administration of saline water can intensify salinity stress and ultimately diminish agricultural yield, thereby jeopardizing food security [7].

Furthermore, in conjunction with the impact of water quality on agricultural output, there exists an increasing focus on the quality and safety of irrigation water in connection with food safety concerns. Irrigation water serves as both a water resource and a conduit for pathogens, including *Escherichia coli*, *Salmonella*, and *Listeria*, posing health hazards and underscoring the need for effective water management in agriculture [8]. Within irrigation water distribution systems, these pathogens not only exist as planktonic cells but can also form part of complex surface-attached microbial communities, such as biofilms [9]. These biofilms constitute protective extracellular polymeric substance (EPS) matrices that are key to promoting microbial survival, modulating pathogen persistence and release, and controlling hydraulic stability and biogeochemical functioning of the entire system [10].

In many coastal, arid, and intensively farmed regions, farmers increasingly rely on blending marginal-quality saline water (e.g. brackish groundwater, drainage water, or diluted seawater) with freshwater to sustain crop production, making “irrigation-relevant” salinity a realistic operating condition rather than a theoretical extreme [5, 6, 11]. Under saline or mixed-salinity conditions, biofilms are particularly relevant, as they can influence the retention and release of salts and nutrients, alter pipe hydraulics, and provide physical shelter that allows embedded pathogens to withstand hydraulic shear and chemical stresses. Thus, understanding how saline irrigation regimes reshape irrigation-system biofilms is essential both for crop performance and for microbiological water safety [12–14].

An important environmental factor that is involved in forming the composition and activities of biofilms is salinity, producing selective environments promoting halotolerant or halophilic populations and thereby modulating community structures [15, 16]. However, most existing work has focused either on bulk-water communities or on simplified mono- or few-species biofilms, often under salinity conditions that are not directly representative of blended irrigation water [17, 18]. As a result, it remains unclear how irrigation-relevant salinity affects the coupled structural, mechanical, and functional dynamics of complex multispecies biofilms growing on pipe surfaces, and how these changes may influence pathogen retention and release during irrigation [19–21].

To address these gaps, we designed a controlled annular biofilm reactor (ABR) system as a laboratory proxy for an irrigation water distribution pipe, operated with an artificial freshwater source and a representative blended salinity (0.6% NaCl) previously identified as agronomically optimal for certain salt-tolerant crops [22–25]. We hypothesized that long-term exposure to this irrigation-relevant salinity would (i) suppress cellular accumulation while stimulating EPS production, leading to a decoupling between cell and matrix growth, and (ii) restructure the multispecies community towards a less diverse but functionally specialized state. By combining high-resolution confocal microscopy, atomic force microscopy (AFM)-based nanomechanics, 16S rRNA gene sequencing, and metatranscriptomics, we aimed to obtain an integrated mechanistic picture of how saline irrigation regimes reorganize biofilm architecture, mechanics, and microbial function in a simulated irrigation piping system. This work builds on a previously established annular reactor model for irrigation biofilms, in which the same artificial freshwater source and reactor configuration were shown to generate reproducible multispecies biofilms with well-characterized biomass, EPS, and community profiles [26], and here we extend this model to explicitly resolve salinity-driven changes in biofilm structure and microbiome function.

## Materials and methods

### Setup of the multispecies biofilm model and study design

A standardized multispecies biofilm model was established in ABRs with modifications to previously described methods (Fig. S1) [26–29]. An indoor aquarium system was maintained as the artificial freshwater source. Five liters of aquarium water were drawn and transferred into a glass holding tank, connected to a 1 L ABR (BioSurface, USA) via silicone tubing (model 17, LongerPump, UK), with recirculation driven by a peristaltic pump (BT100-1 L, LongerPump, UK). In the holding tank, aquarium water was supplemented with 1 mM sodium citrate as an additional carbon source and replaced twice a week to maintain consistent nutrient availability and prevent metabolic accumulation [30].

Two ABR systems were operated in parallel: one control group and the other treated with 0.6% NaCl. The 0.6% salinity level, identified as the optimal blending threshold for irrigation, has been shown to significantly enhance the productivity of salt-tolerant crops and was therefore selected as the critical exposure threshold. Both reactors were equilibrated for two weeks before coupon insertion. Twenty sterile stainless-steel coupons were embedded in each ABR to provide surfaces for biofilm harvesting. The physicochemical parameters (e.g. pH, dissolved oxygen, conductivity, and total dissolved solids) of holding tanks and the baseline of the aquarium water were monitored using probes (Pasco Scientific, USA) throughout the study to ensure consistency.

Biofilms were sampled from both systems at weeks 1, 3, 5, and 8. These time points were chosen to span different stages of biofilm development in the annular reactor: week 1 represents early-stage colonization, week 3 a developing biofilm undergoing community succession, week 5 an intermediate stage approaching pseudo-steady state, and week 8 a later-stage, relatively mature biofilms after several weeks of continuous exposure to freshwater or saline conditions in the simulated irrigation piping system. This 1–8-week window is consistent with week-scale biofilm development commonly reported for flow-through reactors and flumes and is intended to mimic the build-up and early maintenance phase of biofilms during a crop irrigation cycle, when blended saline water is applied over several weeks [31–33]. Focusing on this window also avoids artifacts associated with very old biofilms dominated by sloughing and clogging dynamics rather than net growth. For each treatment and time point, biofilms from each independent reactor run were considered biological replicates for downstream analyses (Table S1). In this first mechanistic study, the freshwater biofilm grown in the same annular reactor was treated with the same aquarium source water as the primary internal control, allowing isolate the specific effects of irrigation-relevant salinity without additional variability from different sites or water sources.

### Qualitative and quantitative assessment of biofilms

The six components of the biofilm were stained using paired fluorescent dyes for 10 minutes in three separate sets to prevent overlap of fluorescence signals. Live (membrane-intact) and dead (membrane-compromised) cells were stained with Syto9 and propidium iodide (PI) (L7012, Invitrogen, USA) according to the instruction, lipids and proteins with nile red [10 μg/mL in phosphate-buffered saline (PBS)] and fluorescein isothiocyanate (FITC) isomer (50 μg/mL in PBS), and α-polysaccharides (α-PS) and β-polysaccharides (β-PS) with concanavalin A tetramethylrhodamine (ConA-TMR) conjugate (1 mg/mL in PBS) and calcofluor white M2R (CFW, 250 μg/mL in PBS), respectively. The Live/Dead assay (Syto9/PI) was used here as a structural indicator of cell-associated biovolume and membrane integrity, rather than as an exact enumeration of viable and non-viable cells, acknowledging that membrane-compromised or dormant cells may not accurately reflect culturability. The combined confocal laser scanning microscope (CLSM) and multiplex-staining approach has been previously applied and benchmarked in similar multispecies biofilm models, providing robust quantitative estimates of biomass and EPS composition [34, 35].

Biofilm samples were imaged using a CLSM (Zeiss LSM 980, Germany) with details of the dye channels and microscope settings listed in Table S2 [26]. For each sampled biofilm, random fields of view were first imaged using a 10× objective to assess overall coverage and uniformity, followed by acquisition of z-stacks at 1 μm intervals using a 40× objective in representative regions. The resulting CLSM datasets enabled a qualitative comparison of biofilm architecture using Fiji and quantitative analysis with the Auto-PHLIP-ML (V1.0.1) toolbox in MATLAB (R2023b), yielding morphometric descriptors including the total biovolume (μm^3^), surface coverage (%), mean thickness (μm), and biofilm roughness (dimensionless) [36, 37]. Considered together for both cellular and EPS fractions, these CLSM-derived metrics serve as integrated quantitative proxies for general biofilm biomass and matrix production under freshwater versus saline conditions.

For each coupon, morphometric descriptors were first averaged across 5–7 randomly acquired z-stack fields of view, and the resulting coupon-level means were treated as individual biological replicates in comparisons between freshwater and saline treatments (Table S1). Statistical differences between freshwater and saline biofilms at each sampling time were assessed using unpaired two-sample Student’s *t*-tests (two-sided) in OriginPro 2025, with *P* < .05 considered statistically significant [38].

### Biofilm mechanical study

The elastic and adhesive properties of biofilms were evaluated using AFM by analyzing indentation and retraction curves [39, 40]. Measurements were conducted with a JPK NanoWizard 4 BioAFM (Bruker, Germany) equipped with a Bruker NP-O cantilever (B-type, USA) bearing a 10 μm borosilicate probe. Biofilm-coated coupons were mounted in a JPK liquid cell holder and submerged in a 0.1 M NaCl solution, and measurements were focused on the biofilm-liquid interface. For each coupon, three force maps were acquired at spatially separated locations; each map consisted of a 4 × 4 array of force curves (16 curves) over a 10 × 10 μm^2^ area. Force curves were collected at a rate of 1 μm/s to minimize hydrodynamic effects, with indentation limited to a force set-point of 9–11 nN.

Force-indentation curves were analyzed using the JPK Data Processing software (v7.0.153, Bruker). The approach segments of the curves were fitted with the Hertz contact model for a rigid spherical indente on an elastic half-space, which is the default model implemented for the NP-O colloidal probe. In this framework, the relationship between the applied force F and indentation depth δ is


$$ F=\frac{4}{3}{E}^{\ast }{R}^{1/2}{\delta}^{3/2} $$

where R is the probe radius, and ${E}^{\ast }=\frac{E}{1-{\nu}^2}$ is the reduced Young’s modulus, with E the apparent Young’s modulus of the sample and ν the Poisson’s ratio. For soft, water-rich biofilms the sample was assumed to be incompressible and ν = 0.5 [41, 42]. The JPK software determines E for each force curve by non-linear least-squares fitting of this equation, using the known probe radius and deflection sensitivity.

Adhesion forces were obtained from the minimum of the retraction branch of each force-indentation curve (pull-off force) as reported by the JPK software. For each coupon, Young’s modulus and adhesion force were averaged over 48 force curves (three 4 × 4 force maps), and these coupon-level means were used as biological replicates for each treatment at week 8 (Table S1). Given the small number of independent reactor runs (*n* = 2 per treatment), AFM-derived mechanical parameters are reported as mean ± standard deviation, and no formal hypothesis testing was performed.

For each coupon, Young’s modulus and adhesion force were calculated from the corresponding force maps by averaging across the 3 spatially distinct locations, yielding one coupon-level value per reactor run. These coupon-level values were used as biological replicates for each treatment at week 8 (Table S1). Given the small number of biological replicates (*n* = 2 per treatment), AFM-derived mechanical parameters are reported as mean ± standard deviation and interpreted descriptively, and no formal hypothesis testing was performed. All experiments were independently duplicated for each biofilm growth condition.

### DNA extraction, 16S amplicon sequencing analysis

For 16S rRNA gene sequencing, samples were collected at weeks 1, 3, 5, and 8 from both freshwater and saline reactors, matching the time points used for the structural biofilm analyses (Table S1). For each treatment and sampling time point, biofilm biomass from three coupons within the same reactor run was pooled and used to construct one 16S rRNA gene library, ensuring sufficient DNA yield for high-throughput sequencing. Two independent reactor runs per treatment were sampled in this way, resulting in two biological replicate libraries per treatment at each time point (*n* = 2; 16 libraries in total across both treatments and all time points). These pooled libraries, rather than individual coupons, were treated as biological replicates in all downstream 16S-based analyses (Table S1).

Biofilm samples were collected from coupons using a sterile scraper, sonicated at 28 kHz to detach residual biomass, and suspended in PBS. Suspensions were centrifuged at 4000 × g for 40 min at 4°C to obtain biofilm pellets, which were stored at −20°C until DNA extraction [26]. Genomic DNA was extracted using the ZymoBIOMICS DNA Miniprep Kit (Zymo Research, D4300, USA) according to the manufacturer’s instructions. DNA concentration and purity were assessed with a NanoDrop spectrophotometer (Thermo Fisher Scientific, USA). Extracted DNA samples were transported on dry ice to Novogene (Beijing, China) for 16S rDNA amplicon sequencing and analysis.

The 16S rRNA gene amplicon sequencing was conducted using the V4 hypervariable region with primers 515F (5’-GTGCCAGCMGCCGCGGTAA-3′) and 806R (5’-GGACTACHVGGGTWTCTAAT-3′). Library preparation involved PCR amplification, followed by purification, end repair, A-tailing, adapter ligation, and further purification. Quality assessment of the libraries was performed using the AATI Fragment Analyzer to confirm insert size and QPCR to quantify effective concentration. Qualified libraries were pooled in appropriate ratios and sequenced on the Illumina NovaSeq platform using a PE250 strategy [43].

Bioinformatic processing was carried out using the QIIME2 pipeline (version 2022.2) [44]. Raw paired-end reads were demultiplexed, and primers were removed. Sequences were quality filtered, denoised, and merged using DADA2 to obtain amplicon sequence variants (ASVs) [45]. Chimeric sequences were identified and removed using the VSEARCH algorithm against the SILVA 138.1 database [46, 47]. Taxonomy was assigned to ASVs using a pre-trained Naive Bayes classifier based on the SILVA 138.1 reference database to determine microbial community composition at 99% sequence identity. Alpha diversity indices (Chao1, Shannon, Simpson, etc.) and beta diversity metrics (weighted/unweighted UniFrac, Bray-Curtis) were calculated. Principal Coordinates Analysis (PCoA), Principal Component Analysis (PCA), and Non-metric Multidimensional Scaling (NMDS) were performed to visualize community differences. Group differences in community structure and taxon abundances were assessed using statistical methods, including ANOSIM, Adonis, LEfSe, and MetagenomeSeq at a significance level of *P* < .05. Raw 16S rRNA gene amplicon reads have been deposited in the NCBI Sequence Read Archive under BioProject PRJNA1334309.

### RNA extraction, meta-transcriptomic sequencing analysis

Metatranscriptomic analysis was performed on week-8 biofilms from both treatments, as this later-stage, relatively mature state is well-suited for assessing near steady-state functional adaptations and aligns with multi-week sampling schemes commonly used for metatranscriptomic biofilm studies in flow-through reactors [48]. For each treatment at week 8, biofilm biomass from five coupons was pooled to obtain sufficient RNA, and one RNA library per treatment was prepared from the pooled material (Table S1). Biofilm biomass was removed from the coupons using a sterile pre-frozen scraper and suspended in PBS. The suspensions were centrifuged at 5000 × g for 3 min to obtain biofilm pellets, which were snap-frozen in liquid nitrogen and stored at −80°C [28]. The pellets were sent to Novogene (Beijing, China) for RNA extraction, quality assessment, library construction, and metatranscriptome sequencing and analysis. Total RNA was extracted by standard protocols and subjected to quality control assessments by agarose gel electrophoresis, NanoPhotometer spectrophotometer (Implen, Germany), Qubit 2.0 Fluorometer (Thermo Fisher, USA), and Agilent 2100 Bioanalyzer. Ribosomal RNA was removed using a Ribo-Zero rRNA Removal Kit (Illumina, Inc.) to enrich mRNA, which was then fragmented and reverse-transcribed into cDNA for the construction of sequencing-ready libraries. The qualified libraries were quantified via qPCR and subsequently sequenced on an Illumina NovaSeq 6000 platform with 150 bp paired-end reads [49].

Raw reads were processed to remove adapters, low-quality sequences, and poly-N stretches, yielding clean data. Residual rRNA sequences were identified and filtered out. De novo transcriptome assembly was performed using Trinity (v2.6.6), and the resulting transcripts were deduplicated using CORSET (v1.09) to obtain the longest cluster sequences for downstream analysis. Taxonomic annotation was performed by aligning all rRNA-depleted assembled unigenes against the NCBI NR database (version 2023-03-01, blastx, evalue ≤1e-5) using DIAMOND (v2.1.6) [50]. Taxonomic profiles were derived from these best BLAST hits and therefore represent the distribution of expressed protein-coding genes across taxa, rather than genome-normalized community composition. Functional annotation was subsequently carried out by comparing unigenes to the GO, Kyoto Encyclopedia of genes and genomes (KEGG), CAZy, and eggNOG databases using the same software and parameters [51].

Gene expression levels were quantified by mapping clean reads from each sample to the Trinity-assembled transcriptome using RSEM (v1.2.28) with Bowtie2 (default parameters, mismatch = 0) to obtain gene-level read counts. These counts were then normalized for library size using the trimmed mean of M-values method implemented in edgeR (v3.28.0). Because one pooled RNA library was generated per treatment, differential gene expression between week-8 freshwater and saline biofilms was evaluated using the edgeR workflow for experiments without biological replication, which models count data with a Poisson distribution and controls the false discovery rate (FDR) using the Benjamini–Hochberg procedure. Genes with an absolute log2(fold change) ≥ 1 and FDR-adjusted q-value <0.005 were considered significantly differentially expressed; positive and negative log2(fold change) values were interpreted as up- and down-regulation in saline biofilms, respectively. Subsequently, enrichment analysis of the differentially expressed genes was conducted using GOseq (v1.32.0) for GO terms and KOBAS (v3.0) for KEGG pathways, with a corrected *P*-value <.05 for both analyses. No metagenomic (DNA-based) sequencing was performed on the same biofilm samples; therefore, taxonomic patterns from the metatranscriptomes would be cross-referenced with the 16S rRNA gene amplicon data interpretation and primarily focus on functional (GO/KEGG) rather than taxonomic conclusions from the RNA-seq data. Raw metatranscriptomic reads from week-8 biofilms have been deposited in the NCBI Sequence Read Archive under the BioProject accession PRJNA1334309.

## Results

### Physicochemical monitoring of the holding tank water

The high reproducibility of the data within the two treatment groups indicated that the biofilm reactors are capable of producing biofilm samples with high repeatability and reliability. The physicochemical conditions of the aquarium water baseline remained stable throughout the study, ensuring a consistent experimental environment (Fig. S2). Against this controlled background, the high reproducibility of the data observed within the two treatment groups indicated that the biofilm reactors are capable of producing biofilm samples with high repeatability and reliability (Fig. S3).

### Qualitative and quantitative assessment of freshwater biofilm models grown in the presence or absence of salt

**Figure 1:**
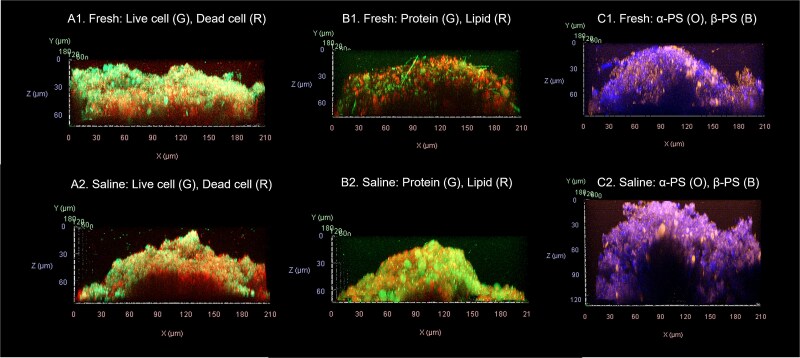
Side view of representative 3D-reconstructed CLSM images (40×) at week 8. (A) Live (Syto9, green) and dead (PI, red) cells, (B) lipids (Nile red, red) and proteins (FITC, green), and (C) α-PS (ConA-TMR, orange) and β-PS (CFW, blue) in freshwater (group 1) and saline water (group 2) biofilms.

The influence of salt exposure on general freshwater biofilm growth and structural development was determined by comparing total cellular and EPS biovolumes, surface coverage, mean thickness, and roughness of biofilms cultivated in ABR systems at weeks 1, 3, 5, and 8 under control and saline treatments. Three-dimensional reconstructions (Fig. 1) revealed clear differences between salt-exposed and control biofilms, which were quantitatively supported by Auto-PHLIP-ML analyses of total biovolume, surface coverage, mean thickness, and roughness for both cellular and EPS components (Table S3).

**Figure 2:**
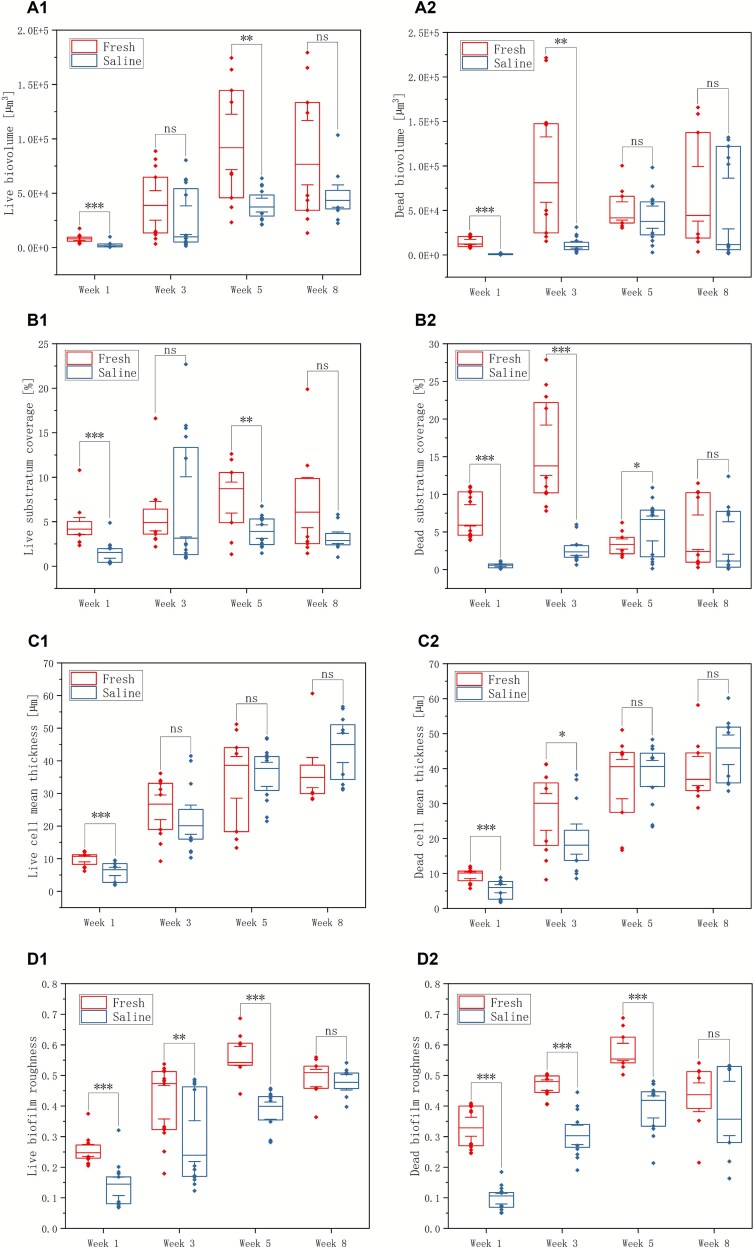
The biovolume (A), substratum coverage (B), mean thickness (C), and roughness (D) of live cells (group 1) and dead cells (group 2) under freshwater and saline water treatments across weeks 1–8. Asterisks indicate statistical significance: ^*^P ≤ .05, ^**^P ≤ .01, ^***^P ≤ .001; “ns” denotes not significant.

**Figure 3:**
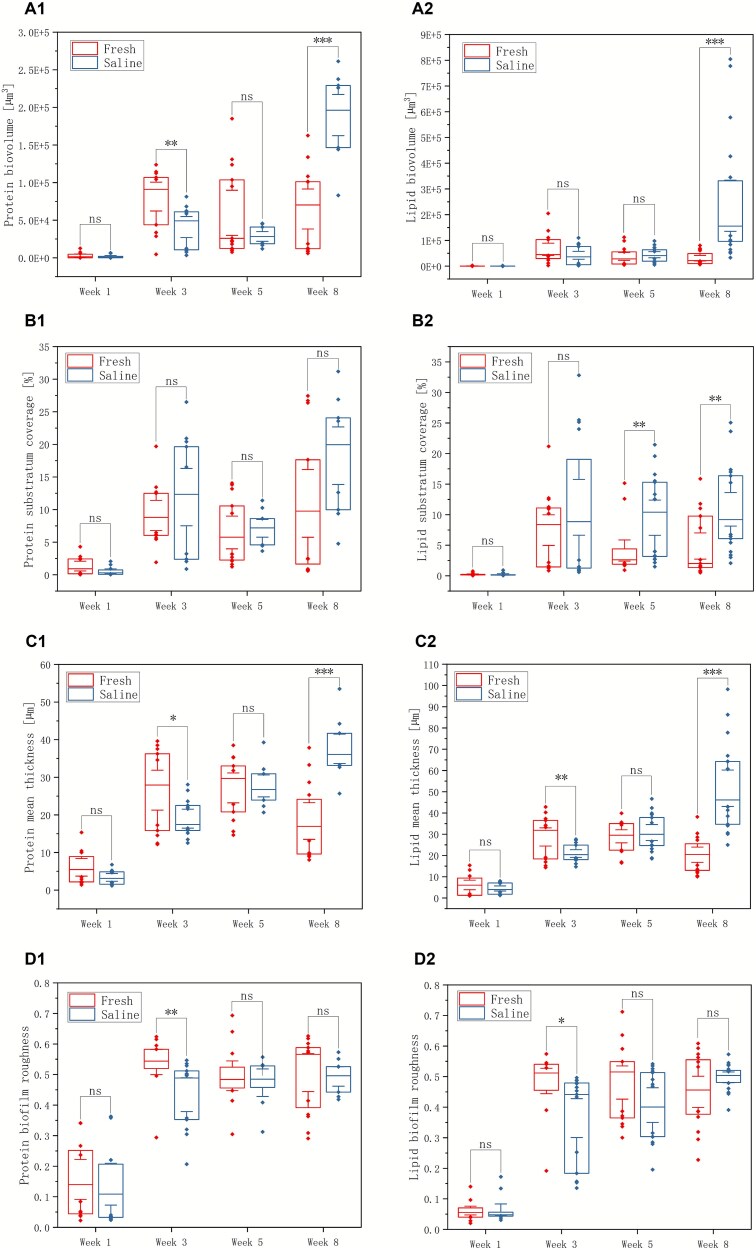
The biovolume (A), substratum coverage (B), mean thickness (C), and roughness (D) of proteins (group 1) and lipids (group 2) under freshwater and saline water treatments across weeks 1–8. Asterisks indicate statistical significance: ^*^P ≤ .05, ^**^P ≤ .01, ^***^P ≤ .001; “ns” denotes not significant.

**Figure 4:**
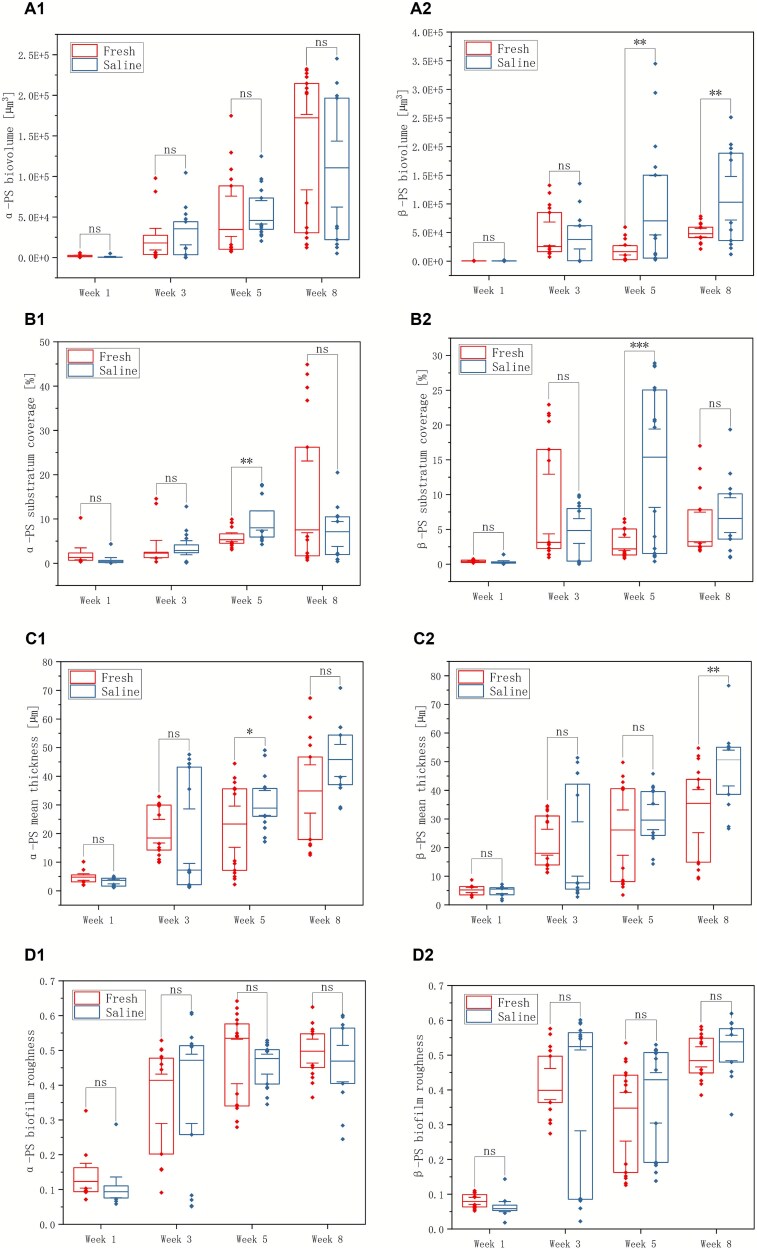
The biovolume (A), substratum coverage (B), mean thickness (C), and roughness (D) of α-PS (group 1) and β-PS (group 2) under freshwater and saline water treatments across weeks 1–8. Asterisks indicate statistical significance: ^*^P ≤ .05, ^**^P ≤ .01, ^***^P ≤ .001; “ns” denotes not significant.

Saline biofilms consistently exhibited lower live (membrane-intact) and dead cell (membrane-compromised) biovolumes than freshwater controls across the 8-week experiment, with the most pronounced differences at the early and intermediate stages. These reductions in cell-associated biovolumes were accompanied by lower cell surface coverage and smaller mean cell-associated thickness in the saline treatment, indicating not only fewer cells but also thinner, less extensive cellular layers (Fig. 2). For the CLSM-based structural analyses, all data points from independent reactor runs are shown in Figs. 2–4 to illustrate biological variability rather than plotting only run-wise averages (Table S3). At week 1, for example, live-cell biovolume was 8034 ± 3449 μm^3^ in freshwater versus 2169 ± 2191 μm^3^ under saline conditions, and dead-cell biovolume was 14 518 ± 5977 μm^3^ versus 903 ± 628 μm^3^, respectively (Table S3). Across weeks 1–3, differences in cell biovolume and coverage, mean thickness between treatments were statistically significant (*P* ≤ .05), except live-cell biovolume at week 3 (*P* > .05). By week 5, mean cell-associated thickness in the saline biofilms began to approach or even exceed that of freshwater biofilms, even though total cell biovolume remained lower, suggesting a transition towards more patchy but locally thicker cell clusters. By week 8, the magnitude of the differences in cell metrics had decreased and no longer consistently reached statistical significance owing to higher variability between reactor runs (Fig. 2).

In contrast, the four EPS components (lipids, proteins, α-PS, and β-PS) showed a delayed but ultimately opposite response to salinity compared with the cellular fractions. At week 1, saline biofilms had similar or slightly lower EPS biovolumes, surface coverage, and mean thickness than freshwater biofilms. From weeks 3 to 5, however, saline biofilms exhibited progressively greater EPS accumulation, with higher EPS biovolume, surface coverage, and mean thickness than freshwater controls (Figs 3–4). By weeks 8, EPS metrics in saline biofilms were frequently higher than in freshwater controls, and the differences in those biovolume, coverage, and thickness were statistically significant for most components (lipids, proteins, and β-PS; *P* ≤ .05), whereas α-PS biovolume in the saline group remained slightly lower than in freshwater, deviating from the overall trend (Figs 3–4). Together, these patterns indicate the development of a thicker, more EPS-dominated matrix under salt exposure, even as total cell biovolume remained lower.

Analyses of roughness and porosity further supported this decoupling between the cellular and EPS fractions. Cell-associated roughness showed comparable variability between treatments and did not differ significantly over time, consistent with a generally patchy but not dramatically restructured cellular landscape (Fig. 2). In contrast, EPS-associated roughness evolved differently, reflecting the gradual build-up of an irregular, polymer-rich matrix under saline conditions (Figs 3–4). Porosity analysis based on cell biomass and EPS roughness revealed no significant differences between saline and freshwater biofilms overall, suggesting that salinity primarily alters the relative contributions of cells and matrix within a broadly similar 3D spatial framework rather than collapsing or radically opening the biofilm structure.

### Biofilm elastic and adhesive properties study

The average and standard deviations of Young's modulus and adhesive force for each replicate (Table 1) revealed considerable variation, which can be attributed to differences in EPS abundance and the heterogeneity of biopolymer molecules on biofilm surfaces. At week 8, freshwater biofilms exhibited a higher average Young’s modulus (463.30 ± 347.46 Pa) than saline biofilms (171.84 ± 163.25 Pa), and a higher average adhesive force (1.69 ± 2.04 nN vs. 0.70 ± 0.48 nN).

**Table 1:** Mean adhesive force and Young’s modulus.

	Young’s modulus (Pa)	Adhesive force (nN)
Week 8, fresh	463.30 ± 347.46	1.69 ± 2.04
Week 8, saline	171.84 ± 163.25	0.70 ± 0.48

This study supports the composite material concept by applying a composite elastic modulus, whereby both rigid cells and the softer EPS matrix jointly contribute to the overall modulus value. Biofilms grown in the absence of NaCl exhibited higher Young’s modulus, likely reflecting a greater proportion of rigid cells and comparatively lower amounts of surface EPS. In contrast, NaCl-grown biofilms displayed more abundant and homogeneously distributed EPS, leading to lower modulus values. Adhesive force measurements further demonstrated reduced adhesion in NaCl-grown biofilms, suggesting a less adhesive surface layer. These interpretations are consistent with microscopic observations of cells and EPS distribution (Fig. 2). Given the small number of independent reactor runs (*n* = 2 per treatment), these mechanical parameters are interpreted descriptively and reported as coupon-level mean ± standard deviation, and no formal hypothesis testing was performed.

### Biofilm community diversity analysis

**Figure 5:**
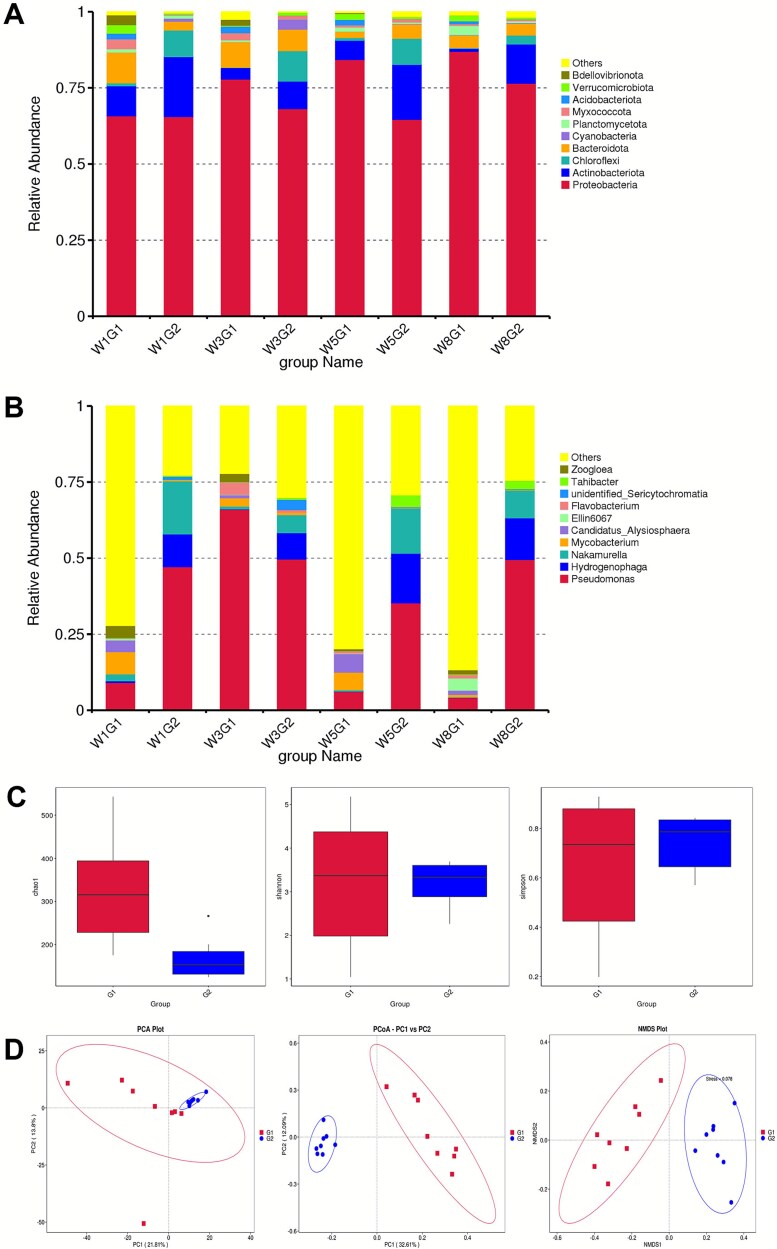
Compositional analysis of modeled multispecies biofilms under freshwater (G1) and saline water (G2) treatments at weeks 1, 3, 5, and 8 (W1, W3, W5, W8). Relative abundance of the top 10 most abundant microbes for 16S rRNA sequences across different treatments and time points. (A) Histogram of relative abundance at the phyum level; (B) histogram of relative abundance at the genus level; (C) boxplot diagram of α-diversity results (Chao1, Shannon, Simpson) between two groups; (D) Beta diversity analyses, including PCA, PCoA, and NMDS displayed consistent diversity distributions among all the samples.

To assess the effects of salt exposure on microbial community composition, 16S rRNA gene sequencing was conducted. Sequence data were analyzed to determine the relative abundances of bacterial taxa at the phylum and genus levels. At the phylum level, *Proteobacteria* predominated across all groups, whereas *Actinobacteria* and *Chloroflexi* were comparatively enriched under saline conditions (Fig. 5A). At the genus level, *Hydrogenophaga* and *Nakamurella* were strongly enriched in saline biofilms but were nearly absent in freshwater biofilms (Fig. 5B).

Saline biofilms exhibited lower species richness and overall diversity, whereas freshwater biofilms maintained comparatively higher community diversity. Relative to the control group, saline treatment significantly reduced microbial α-diversity, as indicated by decreases in both Chao1 richness and Shannon diversity indices, while Simpson’s index suggested slightly higher evenness under saline conditions, reflecting a simplified community structure dominated by fewer taxa (Fig. 5C). Beta diversity analysis revealed significant differences in microbial community composition between the two groups (*P* < .01), and ANOSIM statistics were consistent with this separation. Consistent separation of the groups was observed across PCA, PCoA, and NMDS, visually confirming the statistically supported divergence in community structure between freshwater and saline treatments (Fig. 5D). These diversity patterns mirror the lower live-cell biovolumes and reduced cellular accumulation observed by CLSM in saline biofilms, supporting the consistency between staining-based and sequencing-based assessments of microbial abundance and community simplification.

### RNA-based characterization of the active microbiome and functional responses

**Figure 6:**
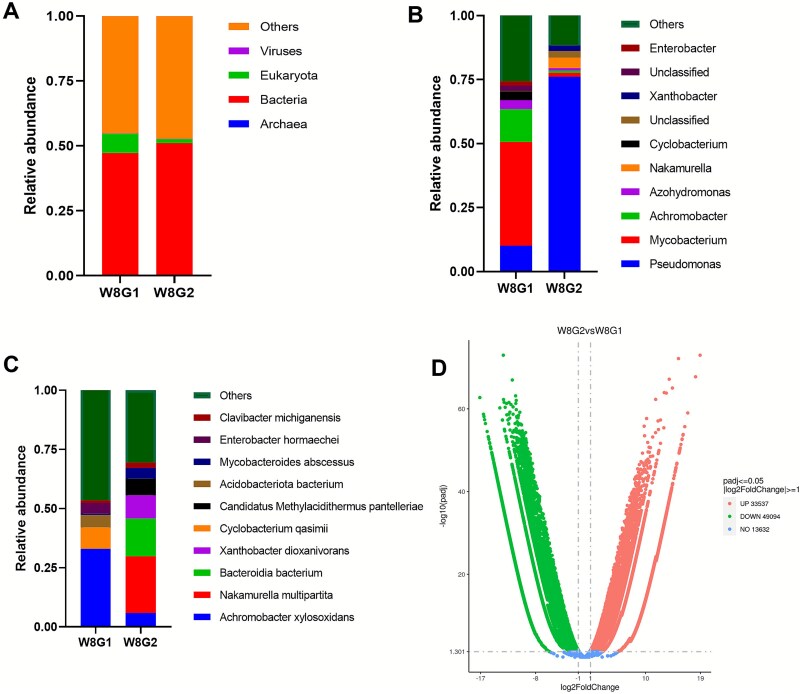
Determination of the active microbiome, differential expression analysis under freshwater (G1) and saline water (G2) treatments at week 8 (W8). Relative abundance of the most abundant active microbiome across treatments and time points at the (A) kingdom, (B) genus, and (C) species levels. (D) Volcano plot of differentially expressed genes between freshwater and saline water groups.

To further investigate the active microbial community and its functional responses to salinity, meta-transcriptomic analysis was conducted. After rRNA removal, taxonomic annotation of protein-coding unigenes showed that, at the kingdom level, bacterial transcripts dominated in both treatments, with slightly higher abundance in the saline group; whereas non-bacterial (Eukaryota) transcripts contributed a somewhat higher expression-weighted fraction of the active community in freshwater biofilms than in saline biofilms (Fig. 6A). Because these proportions are derived from the expressed protein-coding genes rather than from DNA-based genome counts, Fig. 6A should be read as an expression-weighted snapshot of the active microbiome. It provides qualitative context that complements, but does not replace, the 16S rRNA gene-based community composition. At the genus level, *Pseudomonas* was more abundant in saline biofilms (Fig. 6B). Both genus- and species-level analyses revealed that *Nakamurella* was markedly enriched in saline biofilms but nearly absent in freshwater (Fig. 6B and C), a pattern consistent with 16S sequencing results. Using thresholds of |log2(fold change)| ≥ 1 and FDR-adjusted q < 0.005, differential gene expression analysis identified 82 631 genes as significantly regulated between treatments, of which 33 537 were up-regulated and 49 094 were down-regulated in saline biofilms (Fig. 6D).

**Figure 7:**
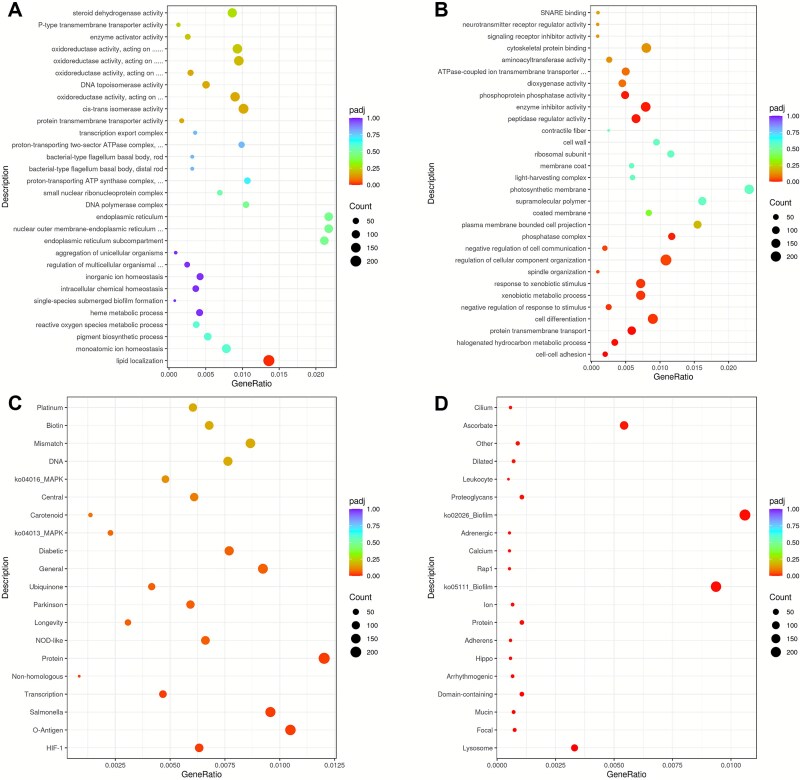
Determination of the enrichment analysis of differentially expressed genes under freshwater (G1) and saline water (G2) treatments at week 8. GO enrichment dot plot showing up-regulated (A) and down-regulated (B) genes in saline water biofilms relative to freshwater biofilms. KEGG pathway-based functional annotation of up-regulated (C) and down-regulated (D) genes in saline water biofilms relative to freshwater biofilms (level 3 pathways).

Gene Ontology (GO) enrichment analysis revealed multiple transcriptional adaptations to saline conditions. Specifically, saline biofilms were characterized by significant up-regulation (corrected *P* < .05) of genes associated with lipid localization, a key function in biofilm adaptation involving cell wall maintenance and potential biosurfactant production (Fig. 7A). Up-regulated genes related to inorganic ion and monoatomic ion homeostasis suggest strengthened ionic regulation under saline conditions. Additionally, elevated expression of genes involved in reactive oxygen species (ROS) metabolism and oxidoreductase activity indicated increased ROS generation, transformation, and detoxification (Fig. 7A). In contrast, genes associated with cell–cell adhesion were significantly down-regulated in saline treatment (corrected *P* < .05), consistent with the observed reduction in biofilm biomass (Fig. 7B).

KEGG enrichment analysis further highlighted functional pathways underlying biofilm responses to salinity. The hypoxia-inducible factor-1 (HIF-1) pathway was significantly up-regulated in saline biofilms (corrected *P* < .05), indicating adaptive responses to low-oxygen conditions (Fig. 7C). Conversely, the down-regulation pathways Ko02026 (*E. coli*) and Ko05111 (*Vibrio cholerae*) suggest reduced intercellular communication and diminished activity of biofilm formation factors in saline environments (Fig. 7D). Collectively, these results highlight the transcriptional reprogramming of microbial communities under saline conditions, reflecting both adaptive mechanisms and constraints on biofilm development.

## Discussion

This research offers a comprehensive mechanistic understanding of the fundamental reorganization processes that multi-species biofilms experience in response to salinity stress, bearing considerable relevance for the management of irrigation water resources. By integrating structural, mechanical, and multi-omics analyses, we demonstrate that salinity triggers an adaptive trade-off: the suppression of cellular proliferation is counterbalanced by a strategic investment in extracellular protection and a functional reassembly of the community. While this response may augment the robustness of biofilms, it could also unintentionally exacerbate the hazards linked with pathogen persistence and the frequency of biofouling within the system.

### Structural decoupling: A trade-off between cellular growth and EPS investment

The most salient finding of this work is the structural decoupling of cellular growth from EPS accumulation. The consistent reduction in both live and dead cell-asscociated biovolumes in saline environments corresponds with the recognized established inhibitory effect of osmotic pressure on microbial proliferation and survivability [16, 19, 52]. In striking contrast, the postponed yet ultimately superior accumulation of all major EPS constituents points to a concerted community-level shift in metabolic resource allocation [53–55]. We posit that this is not a passive consequence of cell death but an active adaptive strategy, whereby the community redirects resources from replication towards the synthesis of protective polymers to buffer against ionic and osmotic fluctuations. Accordingly, we interpret the Syto9/PI CLSM data as describing the total cell-associated biovolume (integrating bacterial and eukaryotic cells with predominantly intact or compromised membranes), whereas the taxonomic and functional partitioning of this active assemblage is resolved by 16S rRNA gene sequencing and metatranscriptomic analyses. The genomic data we collected and analyzed show increased genes related to lipid distribution and ionic balance regulation, supporting this understanding of the biological phenomena.

### Mechanistic origins of altered biofilm biomechanics

This profound structural reorganization directly governs the observed mechanical properties. The significant reduction in Young's modulus and adhesive force in saline biofilms aligns perfectly with the composite material model of biofilms, wherein mechanical properties emerge from the interplay between rigid bacterial cells and a softer EPS matrix [39]. The salt-induced reduction in cell density, alongside a more hydrated EPS gel, alters the mechanical properties towards a softer and less cohesive material. The reduced adhesion suggests a surface that is less amenable to the initial attachment of new cells but which may also facilitate the sloughing off of larger, EPS-bound clusters. The considerable variation in modulus and adhesion highlights EPS heterogeneity, consistent with prior studies on bacterial surfaces [56]. A possible explanation comes from studies showing that biofilms respond non-monotonically to NaCl, being weakened at low and high concentrations but stiffened at intermediate levels [57]. This has been linked to initial EPS stimulation followed by disruption of cell-EPS interactions at higher salt concentration. While our system mainly showed reduced adhesion, such ionic effects on EPS-cell coupling may account for this outcome. It is also worth noting that in other contexts, increased EPS has been associated with stronger adhesion, indicating that the link between EPS and surface adhesion is environment-dependent [39].

### Ecological restructuring and functional specialization

Concurrent with these physico-mechanical changes was a profound ecological restructuring. The decrease in Syto9/PI-stained live and dead cell-associated biovolumes in saline biofilms is mirrored by a reduction in 16S-based richness and Shannon diversity, indicating that independent structural (CLSM) and taxonomic (amplicon sequencing) metrics converge on the same picture of a simplified, lower-biomass community, rather than reflecting a method-specific artifact. The reduction in species richness and Shannon diversity indicates that salinity acts as a strong environmental filter, eliminating salt-sensitive taxa. This loss of ecological complexity likely diminishes the functional redundancy of the community [58, 59]. This filter favored halotolerant experts like *Hydrogenophaga* and *Nakamurella*. The convergence of 16S-based taxonomic profiles, literature reports on the hydrocarbon-degrading capacity of these genera, and the metatranscriptomic enrichment of hydrocarbon degradation pathways indicates that these taxa are not merely tolerant survivors but active key players occupying a specialized niche within the stressed ecosystem [14]. This indicates that salinity does not simply depress microbial function but rather shifts the community towards a more specialized metabolic state [59, 60].

### Transcriptional underpinnings of a stress-adapted state

The metatranscriptomic data provide the crucial molecular underpinnings for these observed phenotypes. The upregulation of genes involved in ROS detoxification and ionic homeostasis represents a direct mechanistic reaction to the osmotic and ionic strain inflicted by salinity [61, 62]. Furthermore, the considerable downregulation of genes associated with intercellular adhesion and traditional biofilm development pathways provides a transcriptional explanation for the diminished cellular biomass and adhesion strength [63, 64]. Because metatranscriptomic taxonomic profiles are inherently influenced by differences in gene expression and gene copy number between taxa, greater emphasis on functional (GO/KEGG) shifts was placed when interpreting the RNA-seq data and the 16S rRNA gene amplicon dataset was used as the primary reference for DNA-based community composition. Together, these patterns suggest that under salt stress, the classical, coordinated developmental pathway of biofilm formation is compromised and replaced by a survival strategy that prioritizes individual cell protection within a communal EPS shelter [65]. The upregulation of the HIF-1 hypoxia pathway further reveals an additional layer of stress, potentially indicating oxygen depletion within the thickened EPS matrix of the saline biofilm [66, 67].

### Synthesis and practical implications for irrigation management

The synthesis of these findings presents a paradigm with critical practical implications. The salinity-induced biofilm condition—distinguished by diminished taxonomic diversity, elevated EPS content, and reduced mechanical properties—may exhibit greater resilience to hydraulic shear and antimicrobial interventions aimed at cellular mechanisms [68]. While the overall microbial load is lower, the enrichment of certain taxa and the protective, sieve-like nature of the EPS matrix could enhance the retention and sheltering of enteric pathogens, should they be introduced into the system [69, 70]. Consequently, the use of blended saline waters, even at levels considered agronomically viable, may promote a biofilm ecosystem that is inherently more stable and protective of embedded microbes [57, 71]. This necessitates a re-evaluation of water management strategies, where salinity is considered not only for its agronomic impact but also for its profound influence on the microbial ecology of the distribution infrastructure itself. Future empirical validation conducted in field settings is imperative to ascertain whether the patterns observed at the laboratory scale are consistent under operational conditions, wherein hydraulic variability, nutrient flux dynamics, and organic matter inputs may interact with salinity stress [72].

### Limitations and future perspectives

While this study provides robust mechanistic insights into biofilm responses to salinity, several limitations must be acknowledged to properly contextualize the findings. First, our laboratory-scale ABR, while commendable for its capacity to facilitate controlled and uniform mechanistic investigations, reduces the multifaceted nature characteristic of authentic irrigation systems. Operational field conditions involve dynamic hydraulic regimes, fluctuating nutrient loads, variable organic matter composition, and seasonal temperature shifts, all of which can interact with salinity to modulate biofilm development in ways not captured here. Consequently, the absolute values of structural and mechanical parameters reported may differ from those in actual irrigation infrastructure. Second, the model biofilm community, though diverse and sourced from a natural freshwater environment, may not fully represent the specific microbial consortia found in all agricultural water sources, particularly those with pre-existing high salinity or organic contamination. Third, the metatranscriptomic analysis at a single time point provides a snapshot of activity but may miss important temporal dynamics in gene expression throughout the biofilm development cycle. Moreover, we did not perform metagenomic (DNA-based) sequencing on the same samples, so taxonomic summaries derived from RNA-seq (e.g. Fig. 6A) should be viewed as expression-weighted activity profiles rather than genome-normalized community structures; to mitigate this, we interpret these RNA-based taxonomic patterns qualitatively and cross-check them against the 16S rRNA gene amplicon data.

In light of these constraints, we deliberately chose in this first study not to include additional field biofilms or alternative water sources as parallel controls, but instead to keep the source water and hydrodynamics constant and vary only salinity within a well-defined laboratory system. This design isolates the specific contribution of irrigation-relevant salinity to biofilm structure and function, at the cost of broader environmental generality. Future work will explicitly extend this framework to biofilms and water samples collected from working irrigation schemes that rely on blended saline and freshwater sources, and to controlled experiments that superimpose realistic shear stresses and nutrient pulses. Introducing relevant bacterial or viral pathogens into this model system would allow for a direct empirical investigation of how these salinity-induced changes in biofilm architecture and mechanics quantitatively affect pathogen incorporation, survival, and release, moving beyond correlation to causation. Over time scales longer than the 8-week window examined here, we expect multispecies biofilms in comparable flow-through systems to enter a quasi-steady, EPS-rich state punctuated by episodic detachment events rather than continuing to grow monotonically. We now explicitly identify this long-term regime as a target for future monitoring in field-scale irrigation networks. Thus, this laboratory model does not represent a final endpoint but rather a critical and necessary foundation upon which more complex, field-relevant studies can be built to ultimately inform safer irrigation practices.

## Summary and conclusion

This study systematically investigates how irrigation-relevant salinity (0.6% NaCl) reorganizes the growth, structure, and function of multi-species biofilms. A controlled ABR model was employed with multi-faceted complementary analyses of structure, mechanics, community composition, and gene expression. Our findings demonstrate that salinity imposes a fundamental trade-off: cellular proliferation is suppressed while protective EPS production is stimulated. This restructuring generates a biomechanically distinct biofilm that is softer and less adhesive. At the same time, the microbial community is ecologically reconfigured, with reduced diversity but enhanced functional specialization for stress tolerance. Crucially, meta-transcriptomic analysis confirms that this reorganization represents an active biological adaptation, characterized by the upregulation of osmotic and oxidative stress pathways and the downregulation of adhesion genes. Collectively, these findings address the three objectives of this study by establishing a controlled model, delineating structural and compositional reorganization, and assessing the ecological consequences for microbial persistence and pathogen interactions.

These findings indicate that even irrigation-relevant salinity promotes resilient and EPS-rich biofilms that may facilitate pathogen shielding and persistence within distribution systems. Consequently, the management of irrigation water quality should go beyond agronomic chemistry to account for microbial ecosystem dynamics. While this controlled model provides a mechanistic foundation for predicting biofilm-related risks, future research should advance toward field validation to determine how hydraulic dynamics and environmental fluctuations interact with salinity to ultimately shape microbial safety and sustainable water use in agriculture.

## Supplementary Material

figS1_ycag001

figS2_ycag001

figS3_ycag001

SI_ycag001

## Data Availability

All raw sequencing data generated in this study have been deposited in the NCBI Sequence Read Archive under BioProject accession PRJNA1334309 (https://dataview.ncbi.nlm.nih.gov/object/PRJNA1334309?reviewer=pfcbscpg664kqm9h0492g1spkd). This includes 16S rRNA gene amplicon sequencing data and metatranscriptomic reads from all treatments and time points.
