# Supplementary figures and images for: Biofilm dynamics under salt exposure: insights from irrigation piping systems
Source: ISME Commun. 2026 Jan 8;6(1):ycag001. doi: 10.1093/ismeco/ycag001 (PMC12887301; doi:10.1093/ismeco/ycag001)

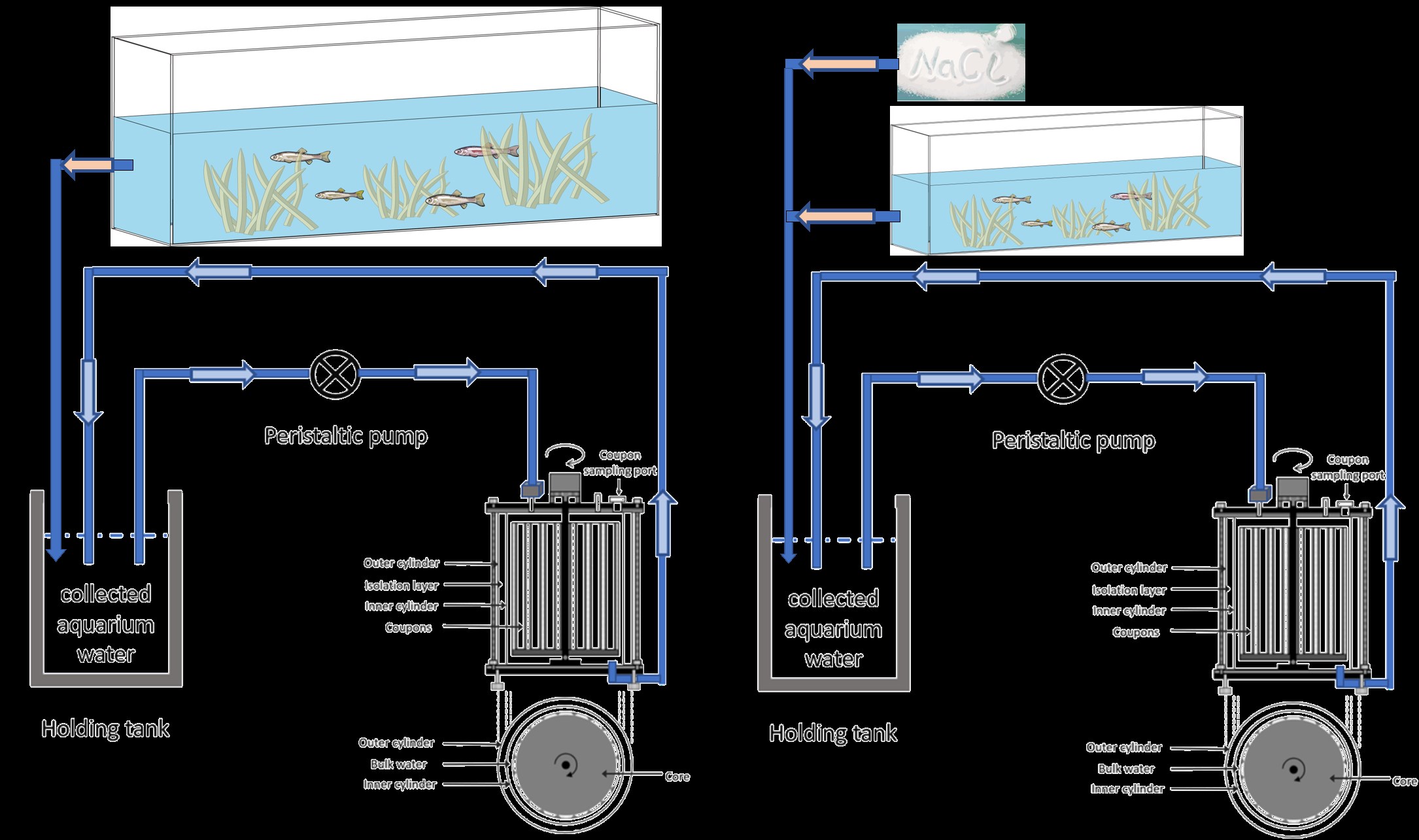

Supplement: figS1_ycag001 [file figs1_ycag001.jpeg]

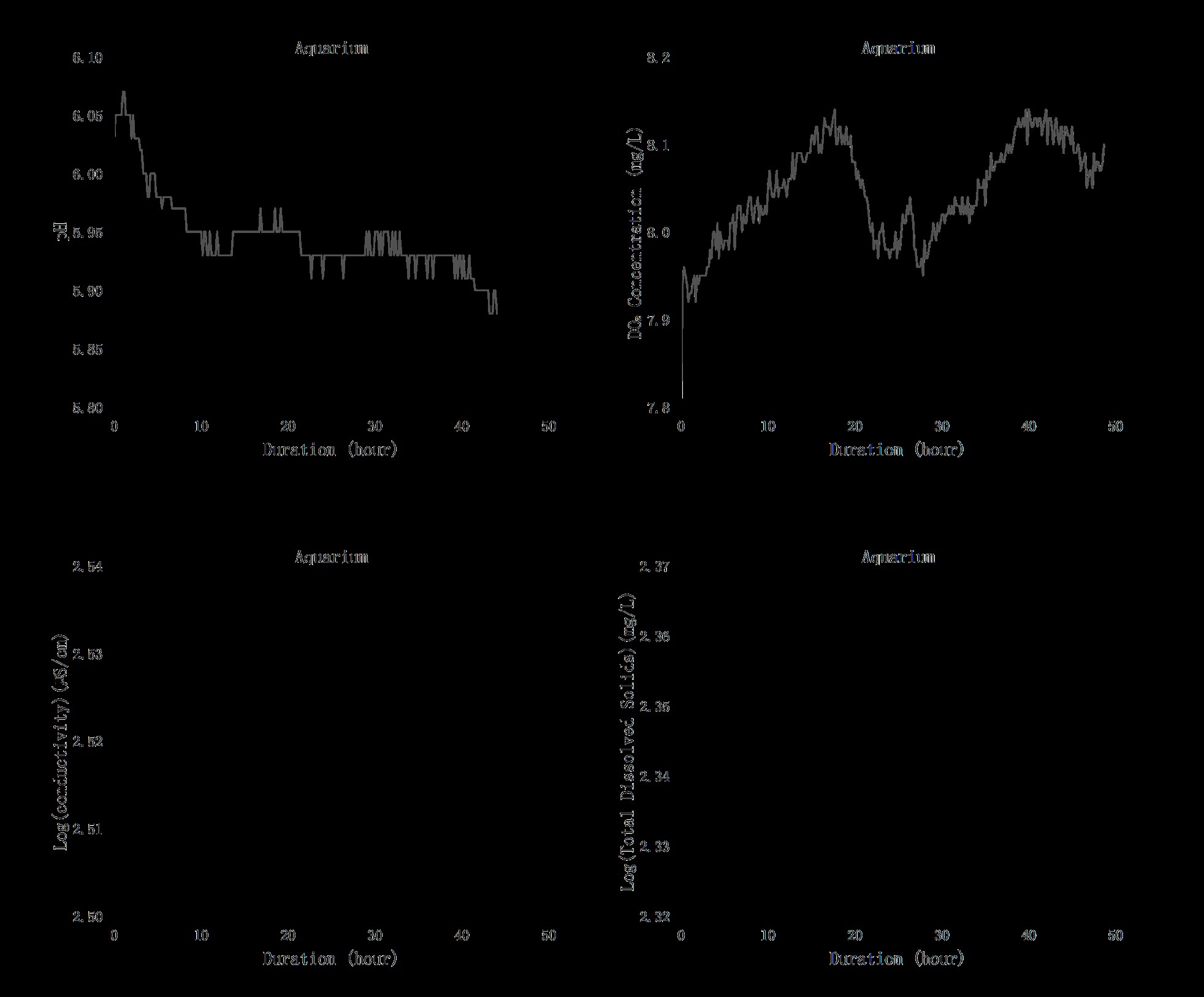

Supplement: figS2_ycag001 [file figs2_ycag001.jpeg]

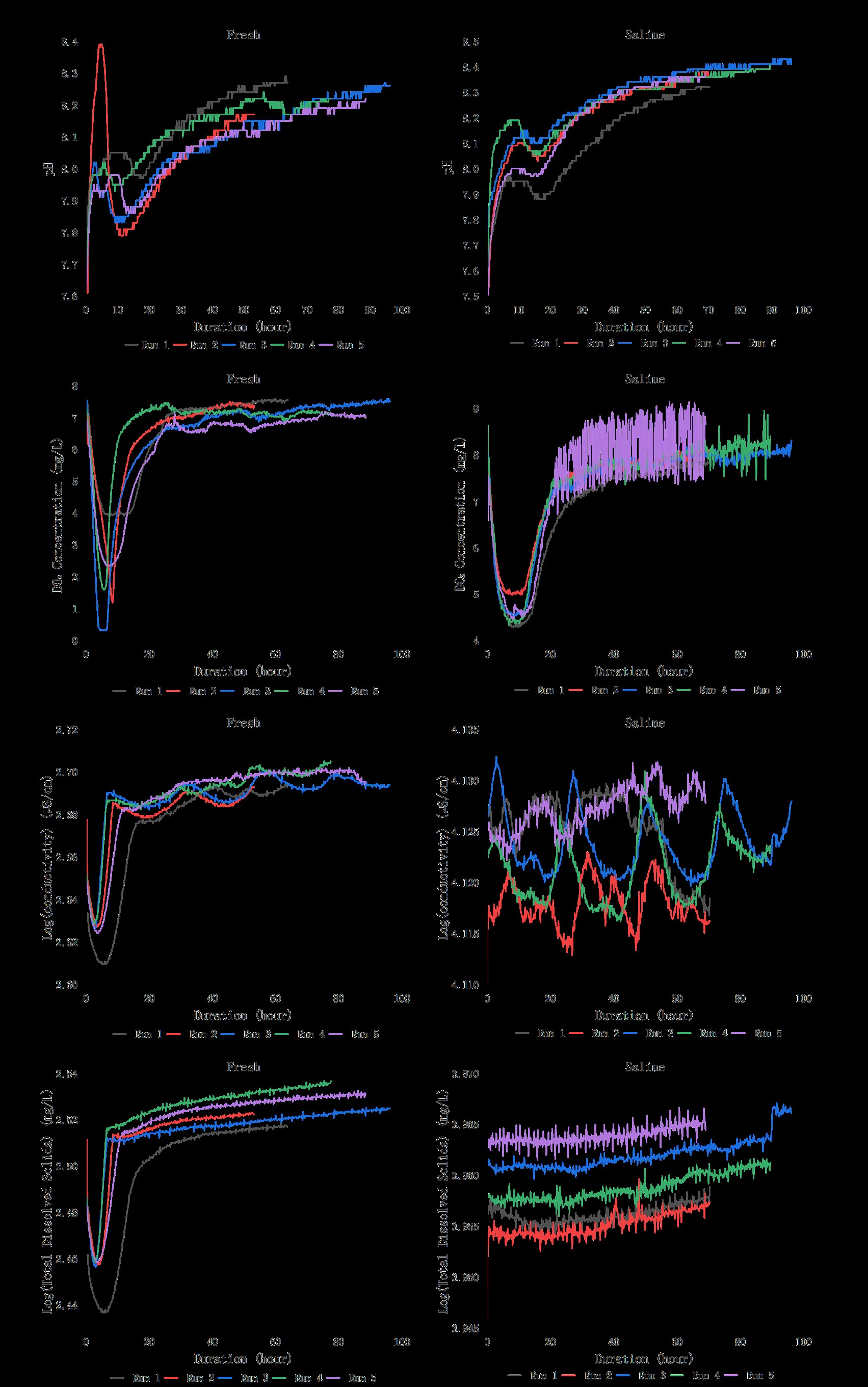

Supplement: figS3_ycag001 [file figs3_ycag001.jpeg]
