# Supplementary material for: Biofilm dynamics under salt exposure: insights from irrigation piping systems
Source: ISME Commun. 2026 Jan 8;6(1):ycag001. doi: 10.1093/ismeco/ycag001 (PMC12887301; doi:10.1093/ismeco/ycag001)
Supplement: SI_ycag001 [file si_ycag001.docx]

**Biofilm Dynamics under Salt Exposure: Insights from Irrigation Piping Systems**

Yan WANG^1,4^, Pengfei HU^2^, Han YU^3^, Alex FURMAN^1,4^, Olivier HABIMANA^3,5*^

^1^ Faculty of Civil & Environmental Engineering, Technion-Israel Institute of Technology, Haifa, 3200003, Israel

^2^ Institute of One Health Science, School of Civil & Environmental Engineering and Geography Science, State Key Laboratory for Quality and Safety of Agro-products, Ningbo University, Ningbo, 315211, China

^3^ Biotechnology and Food Engineering Program; Guangdong Technion-Israel Institute of Technology, Shantou, 515063, China

^4^ Environmental Science and Engineering Research Group; Guangdong Technion-Israel Institute of Technology, Shantou, 515063, China

^5^ Faculty of Biotechnology and Food Engineering, Technion-Israel Institute of Technology, Haifa, 3200003, Israel

*Corresponding author’s contact email: [olivier.habimana@gtiit.edu.cn](mailto:olivier.habimana@gtiit.edu.cn)

SUPPLEMENTARY INFORMATION

**Supplementary** **Figures:**

Figure S1: Schematic diagram of the ABR system: (A) freshwater treatment (control); (B) saline water treatment.

Figure S2: The physicochemical properties baseline of aquarium water just after water change: (A) pH, (B) DO2, (C) conductivity, and (D) total dissolved solids.

Figure S3. Physicochemical properties of holding tank water across five independent rounds: (A1, A2) pH, (B1, B2) dissolved oxygen (DO₂), (C1, C2) conductivity, and (D1, D2) total dissolved solids, under freshwater (left) and saline water (right) treatments, separately.

**Supplementary Tables:**

Table S1: Overview of experimental design and replication structure

| Design  (freshwater & saline) | Sampling weeks | Independent reactor runs per treatment | Coupons analysed per run and time point | Within-coupon measurements (spatial subsamples / technical replicates) |
| --- | --- | --- | --- | --- |
| CLSM | Week 1, 3, 5, 8 | 3 | 1 coupon | 5-7 image fields (z-stacks) per coupon |
| Bio-AFM | Week 8 | 2 | 1 coupon | 3 distinct locations per coupon; 4x4 force map per location; 48 force curves per coupon |
| DNA extraction  (16S amplicon) | Week 1, 3, 5, 8 | 2 | Biofilm from 3 coupons pooled into one DNA library | - |
| RNA extraction  (metatranscriptomics) | Week 8 | 1 | Biofilm from 5 coupons pooled into one RNA library | - |

Note: For each assay, the table reports the sampling weeks, the number of independent reactor runs performed per treatment, the number of coupons analysed per run and time point, and the number of measurements acquired on each coupon (image fields or AFM force curves). For sequencing-based assays, biofilm biomass from several coupons was pooled into a single DNA or RNA library as indicated.

Table S2: Details of the dyes and microscopic settings

| Dye | Syto9 | PI | Nile red | FITC | ConA-TMR | CFW |
| --- | --- | --- | --- | --- | --- | --- |
| Target | Live cells | Dead cells | Lipids | Proteins | α-PS | β-PS |
| Pinhole  (μm/1 AU) | 34 | 42 | 43 | 35 | 41 | 32 |
| Excitation wavelength (nm) | 483 | 305 | 559 | 495 | 552 | 254 |
| Emission wavelength (nm) | 500 | 617 | 636 | 519 | 578 | 432 |
| Laser wavelength (nm) | 488 | 561 | 561 | 488 | 561 | 405 |
| Detection wavelength (nm) | 491-556 | 566-728 | 566-753 | 491-577 | 566-703 | 408-561 |
| Detector type | GaAsP-PMT | GaAsP-PMT | GaAsP-PMT | GaAsP-PMT | GaAsP-PMT | GaAsP-PMT |

Table S3. The quantitative analysis of six biofilm components describes the total biovolume (µm^3^), surface coverage (%), mean thickness (µm), and biofilm roughness (dimensionless). Error represents the standard deviation of the mean of at least n = 12 fields of view from three independent experiments for each time point, weeks 1,3,5, and 8.

| Target | Group | Total biovolume (μm3) | Surface coverage (%) | Mean thickness (μm) | Biofilm roughness |
| --- | --- | --- | --- | --- | --- |
| Live cells | FreshW1 | 8034.18±3448.85 | 4.52±1.96 | 9.97±1.96 | 0.25±0.04 |
|  | FreshW3 | 38797.56±28177.45 | 5.62±3.42 | 25.76±7.84 | 0.41±0.11 |
|  | FreshW5 | 97193.92±52818.22 | 7.71±3.61 | 34.94±13.24 | 0.56±0.06 |
|  | FreshW8 | 87278.97±61307.98 | 7.16±5.85 | 36.39±9.57 | 0.49±0.06 |
|  | SalineW1 | 2169.65±2191.36 | 1.43±1.12 | 6.08±2.63 | 0.14±0.06 |
|  | SalineW3 | 25205.95±27216.98 | 6.67±7.02 | 21.95±9.28 | 0.29±0.14 |
|  | SalineW5 | 39070.08±13121.84 | 3.89±1.57 | 35.85±7.67 | 0.38±0.06 |
|  | SalineW8 | 47372.95±21244.29 | 3.20±1.35 | 43.96±9.27 | 0.48±0.04 |
| Dead cells | FreshW1 | 14518.22±5976.87 | 7.21±2.93 | 9.49±1.96 | 0.33±0.06 |
|  | FreshW3 | 95866.74±76310.61 | 15.86±6.94 | 27.59±10.91 | 0.47±0.04 |
|  | FreshW5 | 49417.35±21322.46 | 3.40±1.40 | 36.99±11.64 | 0.58±0.06 |
|  | FreshW8 | 68629.89±63651.63 | 4.96±4.76 | 39.30±8.65 | 0.43±0.10 |
|  | SalineW1 | 902.82±628.39 | 0.54±0.32 | 5.64±2.41 | 0.10±0.04 |
|  | SalineW3 | 11441.96±7801.18 | 2.60±1.47 | 19.84±8.95 | 0.31±0.07 |
|  | SalineW5 | 42456.97±25906.35 | 5.48±3.44 | 38.61±7.68 | 0.40±0.07 |
|  | SalineW8 | 57779.04±59163.28 | 4.20±4.47 | 45.39±8.77 | 0.39±0.14 |
| Proteins | FreshW1 | 3010.41±3935.06 | 1.34±1.41 | 6.06±4.40 | 0.16±0.12 |
|  | FreshW3 | 81419.82±35997.33 | 9.10±4.34 | 26.56±9.96 | 0.54±0.08 |
|  | FreshW5 | 59899.24±56276.88 | 6.50±4.71 | 27.19±7.45 | 0.50±0.09 |
|  | FreshW8 | 64953.67±49899.85 | 10.95±9.75 | 18.38±9.15 | 0.51±0.12 |
|  | SalineW1 | 1680.23±2320.24 | 0.54±0.66 | 3.37±1.92 | 0.14±0.13 |
|  | SalineW3 | 40900.77±26238.77 | 11.91±8.25 | 19.01±4.73 | 0.43±0.10 |
|  | SalineW5 | 28470.98±12075.31 | 7.12±2.54 | 27.71±5.47 | 0.47±0.07 |
|  | SalineW8 | 189840.87±51474.74 | 18.27±8.28 | 37.58±7.37 | 0.49±0.05 |
| Lipids | FreshW1 | 505.07±715.74 | 0.23±0.20 | 6.15±5.06 | 0.06±0.03 |
|  | FreshW3 | 65403.42±53875.32 | 7.90±5.75 | 28.74±9.63 | 0.49±0.09 |
|  | FreshW5 | 37874.39±34400.79 | 4.24±4.13 | 29.06±6.96 | 0.48±0.12 |
|  | FreshW8 | 30837.79±24521.85 | 4.57±4.96 | 20.39±8.07 | 0.45±0.12 |
|  | SalineW1 | 393.78±561.04 | 0.23±0.25 | 4.50±2.72 | 0.06±0.04 |
|  | SalineW3 | 42183.81±34754.19 | 11.90±10.53 | 20.94±4.03 | 0.36±0.14 |
|  | SalineW5 | 44312.70±26832.74 | 9.83±6.69 | 30.81±8.45 | 0.41±0.11 |
|  | SalineW8 | 234812.75±224104.69 | 10.58±6.32 | 51.66±19.29 | 0.50±0.04 |
| α-PS | FreshW1 | 2043.05±1848.72 | 2.13±2.72 | 4.80±2.31 | 0.14±0.07 |
|  | FreshW3 | 22664.19±26758.11 | 3.19±4.00 | 20.83±8.28 | 0.36±0.14 |
|  | FreshW5 | 50853.48±49882.31 | 5.88±2.03 | 22.40±14.47 | 0.47±0.13 |
|  | FreshW8 | 129910.78±93347.42 | 15.00±16.27 | 35.56±16.96 | 0.50±0.07 |
|  | SalineW1 | 790.48±1352.68 | 0.71±1.18 | 3.18±1.48 | 0.11±0.06 |
|  | SalineW3 | 29861.90±28590.99 | 3.52±3.19 | 19.09±19.12 | 0.39±0.20 |
|  | SalineW5 | 55767.46±28869.60 | 9.67±4.37 | 30.69±8.73 | 0.46±0.06 |
|  | SalineW8 | 102888.41±81703.64 | 6.61±5.65 | 45.53±11.26 | 0.46±0.11 |
| β-PS | FreshW1 | 523.62±236.61 | 0.42±0.19 | 5.18±1.80 | 0.08±0.02 |
|  | FreshW3 | 47693.13±41522.19 | 8.64±8.63 | 21.89±9.10 | 0.42±0.09 |
|  | FreshW5 | 19112.23±16740.49 | 2.92±1.92 | 25.23±15.92 | 0.32±0.14 |
|  | FreshW8 | 49719.64±15125.38 | 5.28±4.42 | 32.73±15.11 | 0.50±0.06 |
|  | SalineW1 | 482.95±544.89 | 0.32±0.36 | 4.83±1.77 | 0.06±0.03 |
|  | SalineW3 | 41428.48±40160.25 | 4.77±3.58 | 19.51±19.08 | 0.40±0.23 |
|  | SalineW5 | 97553.44±104073.26 | 13.80±11.32 | 30.66±8.84 | 0.38±0.15 |
|  | SalineW8 | 109865.50±76584.49 | 7.05±5.03 | 47.80±12.58 | 0.52±0.07 |
